# Atypical parkinsonism: an update

**DOI:** 10.1097/WCO.0b013e3283632da6

**Published:** 2013-07-03

**Authors:** Maria Stamelou, Guenter U. Hoeglinger

**Affiliations:** a Sobell Department for Motor Neurosciences and Movement Disorders, UCL Institute of Neurology, Queen Square, London, UK; b Neurology Clinic, Philipps University Marburg, Marburg, Germany; c Second Department of Neurology, University of Athens, Athens, Greece; d Department of Neurology, Technische Universität München; e German Center for Neurodegenerative Diseases (DZNE), Munich, Germany

**Keywords:** atypical parkinsonian disorders, cerebrospinal fluid biomarkers, diagnostic, genetics, therapy

## Abstract

**Purpose of review:**

This update discusses novel aspects on genetics, diagnosis, and treatments of atypical parkinsonism published over the past 2 years.

**Recent findings:**

A genome-wide association study identified new genetic risk factors for progressive supranuclear palsy and new genetic conditions presenting with atypical parkinsonism have been described. The clinical criteria for diagnosis of corticobasal degeneration have been revised, and for progressive supranuclear palsy are under revision. Novel molecular techniques to identify possible biomarkers, as in other neurodegenerative disorders, have started being studied on atypical parkinsonian conditions, and although preliminary results seem promising, further studies are urgently warranted. Therapeutic trials based on disease-specific targets have shown no clinical improvement.

**Summary:**

The knowledge obtained recently on atypical parkinsonian conditions points out the major deficits in this field. With the expanding phenotypical spectrum of atypical parkinsonian conditions, the early identification of patients has become difficult. The inability of conventional methods to identify these disorders earlier and better than clinicians, and the recent failure of promising therapeutic compounds, highlight the fact that the lack of biomarkers is probably the greatest limitation for developing treatments for these disorders. Thus, current and future research in this direction will be crucial.

## INTRODUCTION

Atypical parkinsonian conditions comprise mostly progressive supranuclear palsy (PSP), corticobasal degeneration (CBD), multiple system atrophy (MSA), and dementia with Lewy bodies (DLB). Apart from these sporadic neurodegenerative disorders, atypical parkinsonism has also been described in a variety of other heredodegenerative conditions such as frontotemporal dementia (FTD), Alzheimer's disease, and Perry syndrome. With the recent advent in genetics, these disorders are constantly increasing [1].

Despite decades of research, the cause and pathophysiology of atypical parkinsonian disorders are still unknown. Moreover, the early differential diagnosis of patients with atypical parkinsonism, which would be important for clinical (e.g., prognosis and treatment) and research purposes, is poor. Therapeutic options are still limited. However, in recent years, there have been some new insights into atypical parkinsonian conditions regarding genetic, diagnostic, and treatment aspects, which will be discussed in this review.

## UPDATE ON GENETICS

Recently, the first genome-wide association study (GWAS) has been performed in PSP to identify genes that modify risk for this primary tauopathy. Apart from confirming two independent variants in *MAPT* affecting risk for PSP, this study identified three significant novel signals associated with PSP risk, which may give some insight into the pathophysiology of the disease. One is at *EIF2AK3,* a gene encoding PERK, which is a component of the endoplasmic reticulum (ER) unfolded protein response (UPR). When excess unfolded proteins accumulate in the ER, PERK is activated and protein synthesis is inhibited allowing the ER to clear misfolded proteins. However, tau, which is the primary misfolded protein in PSP, is not expected to traffic through the ER. The second PSP susceptibility gene, *STX6*, encodes syntaxin 6, and it is hypothesized that genetic variation at *STX6* may influence movement of misfolded proteins from the ER to lysosomes via the endosomal system. Finally, the third locus is the *MOBP* gene, which encodes a protein (MOBP) that is produced by oligodendrocytes and is present in the major dense line of central nervous system (CNS) myelin. MOBP is highly expressed in the white matter of the medulla, pons, cerebellum, and midbrain, regions affected in PSP, suggesting that myelin dysfunction or oligodendrocyte misfunction may contribute to PSP pathogenesis [2]. However, further research is needed to understand how these loci are related to PSP pathophysiology. For MSA, small studies in the past have suggested some possible loci, with the strongest being alpha-synuclein variations (SCNA) [3]; however, a GWAS is currently underway.

**Box 1:**
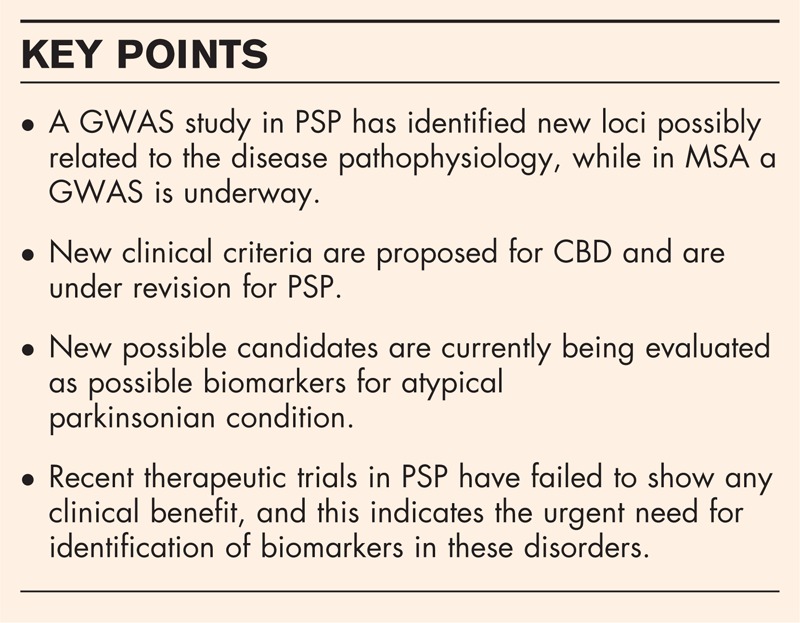
no caption available

Familial MSA has been reported rarely. Whole-genome sequencing and linkage analysis of a sample obtained from a member of a multiplex family in whom MSA had been diagnosed on autopsy revealed a homozygous mutation (M78V–V343A/M78V–V343A) and compound heterozygous mutations (R337X/V343A) in *COQ2* in two multiplex families [4]. *COQ2* encodes parahydroxybenzoate-polyprenyl transferase, which is essential for the biosynthesis of coenzyme Q10. Furthermore, the authors found that a common variant (V343A) and multiple rare variants in *COQ2*, all of which are functionally impaired, were associated with sporadic MSA [4]. This study needs to be further confirmed by independent studies.

In recent years, there has been tremendous research on genetic forms of frontotemporal lobar degeneration (FTLD), which has identified genes that may present with atypical parkinsonism, and in some cases also mimicking PSP, CBD, or MSA [1]. For example, microtubule-associated protein tau (*MAPT*) and progranulin (*PGRN*) gene mutations [tau and TAR DNA-binding protein-43 (TDP-43) pathology, respectively], both inherited in a dominant pattern, are known to cause atypical parkinsonism [1]. However, a major recent breakthrough in the genetics of FTLD and amyotrophic lateral sclerosis has been the identification of hexanucleotide expansions in chromosome 9 open reading frame 72 (*C9ORF72)* gene (TDP-43 pathology), which classically cause FTD–amyotrophic lateral sclerosis overlap syndromes [5^▪▪^,^6^^▪▪^]. However, 35% of these patients may also have atypical parkinsonism, and cases with slowness of vertical saccades, parkinsonism, frontal dementia, and abnormal DaTSCANs, mimicking PSP but also corticobasal syndrome (CBS), have been reported [5^▪▪^,^6^^▪▪^,^7^–^10^]. One study has screened a large population of patients with atypical parkinsonism and found this mutation to be rare in apparently sporadic patients with PSP, CBS, and MSA [11^▪▪^]. In a further study, 3.2% of patients with a PSP phenotype carried the mutation [12^▪▪^]. Apart from the genes causing FTLD, further genetic disorders presenting with atypical parkinsonism have been increasingly recognized [1,13].

## UPDATE ON DIAGNOSTICS

The following section summarizes advances in clinical, imaging, and nonimaging diagnostics and new possible candidates that may serve as biomarkers for these disorders.

### Clinical criteria

Pathology remains the gold standard for definite diagnosis for PSP, CBD, MSA, and DLB. For clinical diagnosis, diagnostic criteria have been widely used; however, these have never been verified prospectively and there is a need for independent clinicopathological confirmation. Recently, a clinicopathological evaluation of the NINDS-SPSP (National Institute of Neurological Disorders and Stroke and Society for Progressive Supranuclear Palsy) [14] vs. NNIPPS (the Neuroprotection and Natural History in Parkinson Plus Syndromes) [15] criteria was conducted on 98 PSP patients and demonstrated that the NINDS-SPSP ‘probable’ criteria yielded shorter time to diagnosis, slightly higher specificity and positive predictive value, and similar sensitivity, compared with the NNIPPS criteria, whereas a combination of NINDS-SPSP possible and probable criteria yielded the highest sensitivity. Thus, it is suggested that the NINDS-SPSP probable criteria might be preferred for recruitment of patients for clinical trials, whereas for routine clinical care, a combination of NINDS possible and probable criteria might be preferred [16^▪^]. However, there is currently a Movement Disorders Society Task Force undertaking revision of the PSP clinical criteria.

Also, new diagnostic criteria for CBD have been proposed. In this study, clinical diagnoses were identified for 267 nonoverlapping pathologically confirmed CBD cases from published reports and brain banks [17^▪^]. Four CBD phenotypes emerged: CBS, frontal behavioral-spatial syndrome, nonfluent/agrammatic variant of primary progressive aphasia, and PSP syndrome, which has also been confirmed by another independent study [18^▪^]. Clinical CBD phenotypes and features were combined to create two sets of criteria: more specific clinical research criteria for probable CBD and broader criteria for possible CBD that are more inclusive but have a higher chance to detect other tau-based diseases [17^▪^]. However, future validation and refinement of the proposed criteria are needed.

### Structural and functional imaging

Several abnormalities in MRI have been proposed in the past as possible candidates to differentiate between atypical parkinsonian syndromes [19]. However, their sensitivity and specificity had not been confirmed in pathologically proven cases. MR images from 48 neuropathologically confirmed cases, including PSP (*n* = 22), MSA (*n* = 13), Parkinson's disease (*n* = 7), CBD (*n* = 6), and controls (*n* = 9) were assessed retrospectively in one study and it was found that the clinical diagnosis was more sensitive in PSP (90.9% in PSP compared with 72.7% in MSA) and the radiological diagnosis more sensitive in MSA (61.5% in PSP compared with 76.9% in MSA) [20]. The radiological diagnosis was more specific than the clinical diagnosis in both PSP and MSA, and radiologically no case of MSA was classified as PSP or vice versa. The most clinically useful abnormalities seen in PSP were signs of midbrain atrophy (86.4%), superior cerebellar peduncle atrophy, and frontal and parietal cortical atrophy. The ‘morning glory flower sign’ and the ‘humming bird sign’ were very specific but with a low sensitivity (50 and 68.4%, respectively). In MSA, only 50% had putaminal atrophy and 25% the hyperintense putaminal rim. Middle cerebellar peduncle hyperintensity was found in 50.0% of the MSA patients, and the hot cross bun sign in 58.3% [20]. These findings suggest that better and novel MRI techniques are needed to improve sensitivity and to be able to serve as specific biomarkers for monitoring disease progression.

Functional imaging has been used in the differential diagnosis of parkinsonism. One recent study showed that sensitivity/specificity of [^18^F]FDG-PET (F-18 fluorodeoxyglucose positron emission tomography) in identifying atypical parkinsonian subgroups was 77/97% for MSA, 74/95% for PSP, and 75/92% for CBD (clinically diagnosed patients) [21]. In another study, 136 parkinsonian patients underwent [^18^F] FDG-PET and the results were compared to a 2-year follow-up of clinical assessment. Concordance of visual evaluation of [^18^F] FDG-PET with clinical diagnosis was achieved in 80% MSA, 76.6% PSP, and 100% CBS patients. Blinded computer assessment using statistical parametric mapping was concordant with the clinical diagnosis in 80% MSA, 93.3% PSP, and 100% CBS patients [22], suggesting its possible usefulness in the differential diagnosis of these disorders.

### Novel diagnostic approaches

Research has focused in recent years on possible novel molecular biomarkers for atypical parkinsonian conditions [23,24]. Studies on specific proteins in cerebrospinal fluid (CSF) have shown some preliminary but often contradictory results: CSF alpha-synuclein levels in MSA have been reported to be decreased compared with controls (but not with Parkinson's disease patients) [24,25^▪▪^], but normal in another study [26]. In PSP and CBD, CSF alpha-synuclein levels were not significantly different compared with controls, but levels in PSP were higher than in Parkinson's disease [25^▪▪^]. CSF total tau and phosphorylated tau levels were not significantly different in PSP and CBD compared with controls in a large study on patients with dementia [27^▪^], but was increased in MSA and CBD compared with Parkinson's disease with no significant change in PSP in another study [25^▪▪^]. Aβ42 did not differ significantly between controls and Parkinson's disease, MSA, PSP, and CBD [25^▪▪^].

Further studies have investigated a combination of proteins. Mollenhauer *et al.*[24] found that CSF mean alpha-synuclein levels and not total tau or Aβ42 levels differentiated Parkinson's disease and MSA from neurological controls (70% sensitivity, 53% specificity), whereas a combination of alpha-synuclein, tau protein, and age discriminated between synucleinopathies and neurological controls and Alzheimer's disease [area under the curve (AUC) 0.908]. Moreover, a combination of alpha-synuclein and phosphorylated tau/total tau could differentiate Parkinson's disease from MSA with a sensitivity of 90% and a specificity of 71% in another study [28]. Neurofilament light chain (NFL) as a biomarker of axonal degeneration was increased in atypical parkinsonian syndromes and differentiated from Parkinson's disease (AUC of 0.93), which has been confirmed in another study [29]. The Flt3 ligand, a cytokine that acts as a neurotrophic and antiapoptotic factor in CNS, could alone differentiate between Parkinson's disease and MSA with a sensitivity of 99% and a specificity of 95% [28]. All these promising approaches need large prospective cohorts of early cases to be confirmed and validated.

Other approaches include the ‘-omics’ (genomics, transcriptomics, proteomics, metabolomics), but less research has been done in atypical parkinsonian disorders in this direction [30^▪▪^]. One study applied a proteomic profiling strategy for parkinsonian diseases using mass spectrometry analysis for magnetic-bead-based enrichment of CSF peptides/proteins and subsequent multivariate statistical analysis on 37 Parkinson's disease patients, 32 MSA patients, and 26 patients with other neurological diseases as controls. Receiver operating characteristics proved that the peak of m/z 6250 was the most important to differentiate MSA from Parkinson's disease, especially in the early stages of the disease; however, these results await confirmation from further studies [31]. Undoubtedly, these methods are promising for the future identification of biomarkers, which are urgently needed in these disorders.

## UPDATE ON TREATMENTS

Although there are still no treatments available for the sporadic atypical parkinsonian conditions, important efforts have been done in recent years, which, even if not proven effective clinically, will certainly guide further research. A randomized, placebo-controlled clinical trial to assess the effects of treatment with the monoamine oxidase-B inhibitor rasagiline (1 mg/day) for 48 weeks in 174 patients with possible or probable MSA-Parkinsonism type, in 39 sites in 12 countries, found no significant difference in progression in the total Unified Multiple System Atrophy Rating Scale (UMSARS) score between the verum and placebo groups [32]. A single-arm, single-center, open-label pilot trial evaluated monthly infusions of 0.4 g/kg intravenous immunoglobulin for 6 months in seven patients as an anti-inflammatory approach, and found significantly increased SBP and improved UMSARS part I (activities of daily living) and II (motor functions); verification in a controlled study was proposed [33]. Lee *et al.*[34] compared 30–50 intraarterial or intravenous injections of autologous mesenchymal stem cells (MSCs, 4 × 10^7^/injection) vs. placebo in 33 patients with probable MSA-cerebellar type and suggested that the MSC group had a smaller increase in total and part II UMSARS scores from baseline throughout a 360-day follow-up period; as the mechanism of action of this intervention remains unclear, a careful experimental and clinical re-evaluation of these findings should be considered [34].

In regard to PSP, a multinational phase 2/3 randomized, double-blind, placebo-controlled trial enrolled 313 participants, to be treated with 30 mg davunetide or placebo twice daily for 52 weeks at 47 sites, and found no significant effect on the co-primary outcome measures, the Progressive Supranuclear Palsy Rating Scale (PSPRS) and the Schwab and England Activities of Daily Living (SEADL) (Press release December 18, 2012 by Allon Therapeutics, www.allontherapeutics.com). An open label pilot trial of lithium, an inhibitor of glycogen synthase kinase-3 (GSK-3), in individuals with PSP or CBD (ClinicalTrials.gov Identifier NCT00703677) recruited 17 patients and was stopped prematurely because the majority of participants did not tolerate the study drug. A multinational, phase II, double-blind, placebo-controlled trial enrolled 142 patients with PSP, who were treated orally with tideglusib (600 or 800 mg p.d.), also a GSK-3 inhibitor, or placebo for 1 year. There were no significant differences between the high dose, low dose, and either dose groups vs. the placebo group in the primary clinical outcome measures. A subset of 37 patients underwent baseline and 52-week MRI; this substudy demonstrated significantly reduced global brain atrophy in tideglusib-treated patients [35]. The effect of GSK-3 inhibition in PSP thus warrants further investigation.

## CONCLUSION

The knowledge obtained recently on atypical parkinsonian conditions points out clearly what are the major deficits in this field, which, compared to other neurodegenerative diseases, lacks major advances in terms of early diagnosis and treatments. With the expanding phenotypical spectrum of atypical parkinsonian conditions, the early identification of patients has become difficult; thus, new clinical criteria are being currently revised. The inability of conventional methods such as MRI to identify these disorders earlier and better than clinicians, and the recent failure of promising therapeutic compounds based on rational therapeutic hypotheses [36] highlight the fact that the lack of biomarkers is probably the greatest limitation for developing treatments for these disorders. Thus, current and future research in this direction will be crucial.

## Acknowledgements

*G.H. is funded by the Deutsche Forschungsgemeinschaft (HO 2402/6-1)*.

### Conflicts of interest

There are no conflicts of interest.

## REFERENCES AND RECOMMENDED READING

Papers of particular interest, published within the annual period of review, have been highlighted as:

▪ of special interest
▪▪ of outstanding interest

Additional references related to this topic can also be found in the Current World Literature section in this issue (p. 453).
